# Supplementary material for: EchoPT: A Pretrained Transformer Architecture That Predicts 2D In-Air Sonar Images for Mobile Robotics
Source: Biomimetics (Basel). 2024 Nov 13;9(11):695. doi: 10.3390/biomimetics9110695 (PMC11591995; doi:10.3390/biomimetics9110695)
Supplement: Supplementary file 1 [file biomimetics-09-00695-s001.zip › biomimetics-3254110-supplementary.pdf]

Supplementary Materials: EchoPT: A Pretrained Transformer Architecture That Predicts 2D In-Air Sonar Images for Mobile Robotics

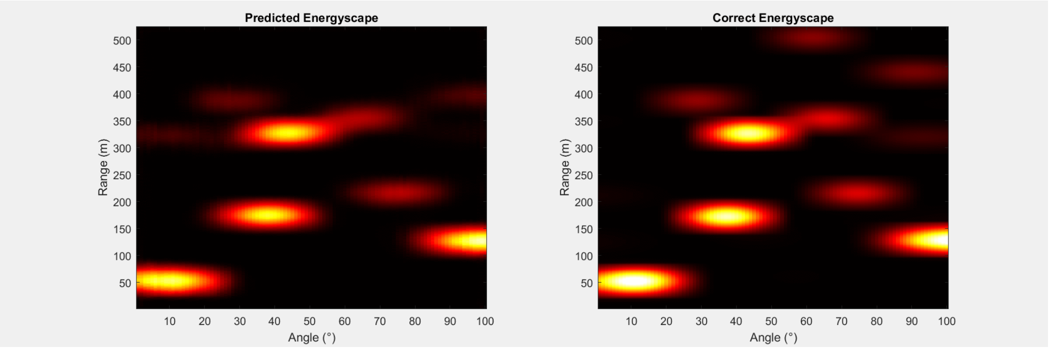

Figure S1. This animation shows the auto-regressive prediction of sonar data using the EchoPT model. To ensure that the animation works, view it with a PDF viewer that supports animations (such as Adobe PDF reader).
